# Does nature-based social prescription improve mental health outcomes? A systematic review and meta-analysis

**DOI:** 10.3389/fpubh.2024.1228271

**Published:** 2024-03-25

**Authors:** Rashid Menhas, Lili Yang, Zulkaif Ahmed Saqib, Muhammad Younas, Muhammad Muddasar Saeed

**Affiliations:** ^1^Department of Nursing, The Fourth Affiliated Hospital of School of Medicine, and International School of Medicine, International Institutes of Medicine, Zhejiang University, Yiwu, China; ^2^College of Urban Transportation and Logistics, Shenzhen Technology University, Shenzhen, China; ^3^School of Educational Technology, Beijing Normal University, Beijing, China; ^4^International Education College Chinese and Western Medicine Clinic, Dalian Medical University, Dalian, Liaoning, China

**Keywords:** nature, social prescription, interventions, mental health, effect size

## Abstract

**Background:**

A nature-based social prescription (NBSP) is an approach to improving mental health outcomes that involves prescribing nature-based interventions as complementary or alternative therapy to traditional ones. A variety of advantages are available from NBSP for people looking to enhance their mental well-being. The effect size of the nature-based social prescriptions (NBSPs) has not been thoroughly evaluated by systematic reviews and meta-analyses.

**Objectives:**

The current study aimed to analyze existing studies and conduct a meta-analysis to determine the overall effect size of the nature-based social prescriptions (NBSP’s) outcomes on mental health.

**Methods:**

By choosing the relevant papers from among those that were available, a meta-analysis was carried out in the current study. A systematic search of electronic databases (Pub Med, Web of Science, Scopus, Cochrane Library, Embase, CINAHL, and PsychINFO) was conducted to identify relevant studies. Studies were included if they evaluated the effects of NBSP on mental health outcomes. Effect sizes were calculated using the random effects model.

**Results:**

Meta-analysis of interventions statistics shows that CBT (SMD −0.0035; 95% CI: [−0.5090; 0.5020]; Tau^2: 0.1011; Tau: 0.318), digital intervention (SMD −0.3654; 95% CI: [−0.5258; 1.2566]; Tau^2: 0.2976, Tau: 0.5455), music intervention (SMD −2.1281; 95% CI: [−0.4659; 4.7221]; Tau^2: 3.4046; Tau:1.8452), and psychological interventions (SMD −0.8529; 95% CI: [0.3051; 1.4007]; Tau^2: 0.1224; Tau: 0.3499) do not significantly impact. The other interventions [social belongingness, communication training, blue intervention, nature-based education, cognitive behavior group therapy (CBGT), social prescribing coordinator, self-help intervention, participatory, organizational intervention, inpatient services, brief diet, internet-based intervention, prenatal intervention, yoga and meditation, ergonomics training program, yoga nidra intervention, and storytelling] highlighted above are significant.

**Conclusion:**

The conclusion of the meta-analysis supports the idea that incorporating nature-based social prescription interventions into mental healthcare plans can effectively complement traditional therapies and improve mental health outcomes.

**Systematic review registration:**

https://www.crd.york.ac.uk/prospero/display_record.php?ID=CRD42023412458, CRD42023412458.

## 1 Introduction

Historically, social prescribing has been used in low-income neighborhoods to help those living with a wide range of issues, including but not limited to mental and physical health issues, financial hardships, social and emotional problems, drug misuse, and chaotic lives (1). Social prescribing is expanding the availability of nature-based therapies for prevalent mental health disorders. There is evidence about how nature may be harmful to people, how humans’ health is intertwined with the health of the natural world, and how humans’ actions in the natural world have been (2–6). In this context, the term “nature” refers to the “living nature” of plants, animals, still and flowing water, characteristics of air and weather, and the landscapes that include them and show the impact of natural formations. Therefore, the phrases “nature” and “natural environment,” which refer to a setting with little to no apparent signs of human presence or influence, have often been used interchangeably (2). Eric Fromm, who coined “biophilia” in the late 1960s, was later refined by EO Wilson. The “biophilia theory” postulates that humans have an innate evolutionary bias toward forming bonds with other organisms and engaging in activities that mimic life (7, 8). People’s “nature contact” encompasses direct experiences with real-world aspects of nature, like plants and animals, and representations of nature, which have been linked to improved mental health and well-being (9, 10).

Hedonic well-being is a state of happiness and contentment correlated with time spent in nature (11). Spending time in green spaces, near trees, and engaging in outdoor pursuits like hiking, gardening, and bird watching increases one’s momentary happiness (12). The connection between spending time in nature and eudemonic well-being has received less attention. It incorporates qualities of good mental health, such as authenticity, finding meaning in life, and prospering in one’s most fundamental responsibilities (13). Different individuals have different perspectives on how they should interact with the natural environment, and the nature-relatedness construct shows these divergences. Human-nature interaction has many facets, which can be expressed via feelings of connectedness to nature (14). Interacting with the natural world is generally appropriate for people. The degree of a person’s sense of personal connection to nature has been linked to happiness and ecological consciousness. The contemporary lifestyles that distance people from the natural world are a typical result that negatively affects human and environmental health (15). Experiencing the natural beauty of forests is said to benefit one’s emotional, physiological, and social health. Forests and green spaces seem to be the focus of most research on the possible health benefits associated with time spent in nature (16, 17). The “nature therapy hypothesis” explains the positive impact of nature on human health. Spending time in nature has a positive impact on mental health and well-being (18–20). Both natural and urban forests improve the quality of life of humans. Disinfectants, blood pressure lowering, anti-asthma, and immune-boosting are only a few medicinal qualities found in different plant groups (21).

### 1.1 Exposure to nature and its impact on health

The positive impact of nature on human health and happiness has been the subject of empirical study in various academic fields, with researchers coming to the same conclusion: spending time in natural settings improves people’s emotional and physical well-being (22). Trees, parks, woods, community gardens, grassy verges, and green roofs are all examples of urban green infrastructure that provide a wide variety of experiences to city dwellers (23). Experiencing nature has sound effects on people’s social lives, mental health, and moods and speeds up the body’s healing process (24). People’s attention is renewed, mental tiredness is alleviated, and tension is relieved thanks to nature’s natural eco-environmental factors, which also restore focus (25). Based on this understanding, nature-based therapies encourage individuals to engage in healthy behaviors by engaging in natural activities like gardening, farming, exercise, or interacting with animals (26). Parks’ positive effects on health are conditional on several factors. Larger parks, parks with more green space, and parks with vistas of nature have all been linked to increased happiness among their visitors. It is impossible to overstate how important parks are to human health, and the advantages of visiting parks for health are greatly influenced by the particular park features that visitors like (27). The value of multiple ecological services and functions to people’s health and urban resilience, particularly in the face of public health emergencies, has been demonstrated by recent research on green infrastructure (28). Changes in brain activity in the prefrontal cortex that play a crucial role in emotional regulation have been linked to time spent in nature (29). The increasing expense of chronic diseases has prompted a greater focus on preventative measures, such as prescriptions for spending time in parks that use nature to improve health (30).

The natural and emotional attachment of humans to other living species, defined by biologist Edward O. Wilson, is biophilia. Innate traits are inherited and fundamental to human nature. According to Wilson, biophilia is more accurately described as a “complex of learning norms” refined by thousands of years of evolution and interactions between humans and their environments (31). The process of psychological restoration involves replenishing exhausted resources that have been used up due to adjusting to regular demands, such as stresses and strenuous activities (32). Indoor plants and street trees show how exciting stuff frequently happens in manufactured settings. Allotment (or communal) gardens and urban parks are also created, developed, controlled, and maintained, but they include natural characteristics and provide chances to participate in and follow biological processes. Studies have shown that people may have a genuine “nature experience” even while looking at natural scenes or landscapes from within a structure or a moving vehicle, in a still picture, a moving video, or a virtual reality environment (33).

### 1.2 Nature-based social prescription for health and well-being

Social prescribing has been widely used in the geriatric population to combat isolation, raise rates of physical activity, and enhance general health (34). Culturally and scientifically, human relationships with the natural world are beneficial. In addition, participation and focused therapies involving interaction with nature have grown popular due to the growing relevance of social prescribing in healthcare. The use of nature-based social prescriptions by healthcare professionals is a promising new frontier in assisting people of all ages in fostering a sense of belonging in their communities and the natural world. However, healthcare professionals are also facing challenges regarding the effective prescription of nature for health and well-being (35). The psychophysiological stress recovery theory (PSRT) and Kaplan’s attention restoration theory (KART) are the two main schools of thought discussing what constitutes a therapeutic environment. The PSRT, grounded on the biophilia theory, postulates that people have an inbuilt affinity for natural surroundings because of the benefits these settings provided to their ancestors (2). There are advantages to both the individual and the group when people work on improving their health as part of a community. Two separate studies find that social support under social prescription significantly improves both psychological health and the ability to control chronic medical illnesses (36). Numerous demographic, observational, empirical, and intervention studies have shown the positive health effects of spending time in natural settings, including woods, urban parks, local green spaces, and rural parks. Psychological and physiological health improved after exposure to green spaces (37, 38). One such option is “green exercise prescription,” which involves both movement and time spent in the great outdoors. Contact with nature, such as blue and green areas, indoor plants, and gardening, may attenuate or buffer the adverse health effects, reducing or eliminating them (39). Exposure to more green space under nature-based social prescription has been connected with better mood, perceived overall health, and more significant physical activity (40).

### 1.3 The rationale of the current study

Human health depends crucially on the natural world. Pollination of food crops, water purification, flood prevention, and climatic stability are just a few of nature’s many documented health advantages. Clarifying how nature encourages physical exercise for the various mental and physical health advantages is an essential but challenging study area, especially in highly crowded cities with limited and declining access to natural environments (30). Reducing negative emotions and heightening pleasant ones are all part of the psychological, emotional, and cognitive shifts that occur in nature after a stressful event has ended (31). Park-goers’ physical and mental health may benefit from the variety of nature-, movement-, and community-based recreational activities in a semi-natural setting that satisfies their material and immaterial, solitary, and sociable requirements (41). People have known that spending time in natural outdoor settings is good for mental and physical health (42). Parks, canals, woods, and recreational areas are all examples of natural outdoor habitats, often called green and blue environments or green and blue spaces, since they have aspects of nature such as flora and water (43). Green exercise has been linked to better stress recovery, more remarkable attentional restoration, and a decrease in negative emotions, all of which may aid obese persons in adequately coping with these challenges (42, 44). The emerging subject of lifestyle psychiatry is based on the idea that making positive adjustments to one’s daily habits might help treat mental health problems (45). A stronger connection with nature is good for everyone’s health. However, there is an increasing need for nature-based therapies for mental health outcomes. The effect size of NBSPs has not been thoroughly evaluated by systematic reviews and meta-analyses. Furthermore, while social prescribing is increasingly being used as a non-pharmacological intervention for mental health, there is a lack of research specifically focusing on NBSP’s impact (46). The current study addresses these research gaps by synthesizing the literature on NBSPs and comprehensively evaluating their effect size in improving mental health outcomes.

## 2 Methods

Systematic reviews and meta-analyses greatly aid the synthesis of the evidence. Systematic reviews efficiently synthesize the literature using precise search criteria, followed by a thorough evaluation and logical synthesis of several source studies (47). The reporting of the current review adhered to the Preferred Reporting Items for Systematic Reviews and Meta-Analyses (PRISMA) standards. In-depth and exhaustive search techniques are often used in systematic reviews to help reviewers find all relevant research on a topic (48). Concrete methods for performing a meta-analysis were implemented once the research issue had been specified and particular research questions had been suggested. The extraction of data analysis findings from earlier empirical investigations is a critical component of the research integration process known as meta-analysis. The procedure included a thorough literature search, inclusion and exclusion standards, research coding, effect size analysis, and other steps (49).

### 2.1 The strategy of literature search

Three independent researchers (MY, MS, and ZS) performed the literature search. Literature searches were carried out through eight databases (PubMed, PsychINFO, Web of Science, Scopus, CNKI, Embase, CINAHL, and Cochrane Library). To find pertinent studies, keywords and medical subject headings (MeSH) were combined. The search terms were used (“nature-based social prescriptio*” AND “nature-based interventio*” AND “green car*” AND “ecotherap*” AND “Ecopsycholog*” AND “green exercis*” AND “outdoor therap*” AND “horticultural therap*” AND “green space*” AND “natural environment*” AND “outdoor environment*” AND “natural settin*”) AND (“mental healt*” AND “mental disorder*” AND “mental illness*” AND “depression*” AND “anxiety*” AND “stress*” AND “well-bein*” AND “mental wellness*” OR “mental health outcomes” AND “psychological healt*” AND “emotional healt*” AND “mood disorder*”).

### 2.2 Data extraction

Researcher’s separately extracted data from the chosen papers using a pre-established form after the articles had been selected. Any differences in opinions between the authors were resolved through group discussions. All investigators were involved in evaluating and discussing the final selection of articles. To ensure the accuracy and integrity of the meta-analysis, the data and references from each included study were thoroughly crosschecked by ZAS to prevent duplication.

### 2.3 Exclusion and inclusion criteria (studies selection)

*The inclusion criteria:* (i) the effectiveness of NBSPs for enhancing mental health was examined in studies that were published in English; (ii) studies evaluated the impact of nature-based social prescription, ecotherapy, green therapy, green exercise, and activities performed or related to green spaces or green prescriptions on mental health outcomes; (iii) studies that include a comparison group (control or alternative intervention); (iv) studies that use standardized measures of mental health outcomes such as depression, anxiety, stress, or overall psychological well-being; and (v) studies published between “2017 to 2022” were used.

*Exclusion criteria:* the studies were excluded if (i) do not report original data (e.g., review articles, editorials, and letters); (ii) do not focus on nature-based interventions for mental health; (iii) do not include a comparison group (control or alternative intervention); (iv) do not use standardized measures of mental health outcomes; and (v) animal assisted-based therapy studies. The titles and abstracts of the initial search results were scrutinized for eligibility. The whole text of the included studies was read to evaluate them further. Discussion and agreement among the reviewers were used to settle any disagreements regarding the eligibility of studies.

### 2.4 Statistical analysis

The collected data were analyzed by using STATA 16.0. Publication bias, summary measures, and forest plot and meta-regression were performed. In addition, study characteristics were also assessed.

#### 2.4.1 Heterogeneity

In the meta-analysis, heterogeneity indicates the variation of the outcomes of the studies considered in the analysis. Evaluating heterogeneity is essential in the meta-analysis since various models can lead to various estimates of the overall effect sizes and the different standard errors. The heterogeneity of 0–40% would not be significant, 30–60% indicates a moderate level of heterogeneity, and 50–90% indicates a substantial type of heterogeneity. In comparison, 75–100% reflects considerable heterogeneity in the provided data set (50).

#### 2.4.2 Summary measures and forest plot

Summary measures and forest plots are commonly used in meta-analyses to summarize and present the results of the studies included in the analysis. Summary measures typically include the effect size estimate, the measure of variability (such as standard error or confidence interval), and the weight given to each study in the meta-analysis. A forest plot is a graphical representation of the results of the meta-analysis. The plot displays the effect size estimate, the confidence interval for each study included in the meta-analysis, and the summary estimate for the overall effect size and confidence interval. The forest plot can also display subgroups of studies based on predefined characteristics, such as study design, intervention type, or patient population. Comparing these subgroups will allow you to see any variations in effect sizes.

#### 2.4.3 Publication bias

According to each journal’s predetermined criteria, publication bias is the situation that occurs when specific publishing houses only publish studies with significant results and refuse to publish any articles that have opposing or negligible results. It creates a disturbance in fully explaining the studies in the study area (51). So, the primary cause of publication bias is the only publication of significant or positive results and the exclusion of all other studies that prevent researchers from viewing the complete picture of the research (52). As a result, overestimating the actual effect size leads to misleading interpretations and application of the findings.

#### 2.4.4 Meta-regression

Meta-regression is a statistical technique used in meta-analysis to examine the relationship between a study’s characteristics (such as sample size, age of participants, or publication year) and the effect size observed in the study. However, it is essential to note that meta-regression analysis has limitations and assumptions that must be carefully considered. The equation is given below:


$$Y_{i}=\beta_{0}+\beta_{1}X_{1i}+\beta_{2}X_{2i}+\ldots +\beta_{k}X_{ki}+\varepsilon_{i}$$

Where:

*Y*_i_: the effect size of the ith study.

*X*_1i_, *X*_2i_…, *X*_ki_ is the study characteristics (independent variables) that may be related to the effect size.

*β*_0_ is the intercept, representing the effect size when all independent variables are equal to zero.

*β*_1_, *β*_2_,…, *β*_k_ are the regression coefficients, representing the change in effect size associated with a unit change in each independent variable while holding all other independent variables constant.

*ε*_i_ is the error term, representing the unexplained variability in the effect size not accounted for by the independent variables.

## 3 Results

**Figure 1:**
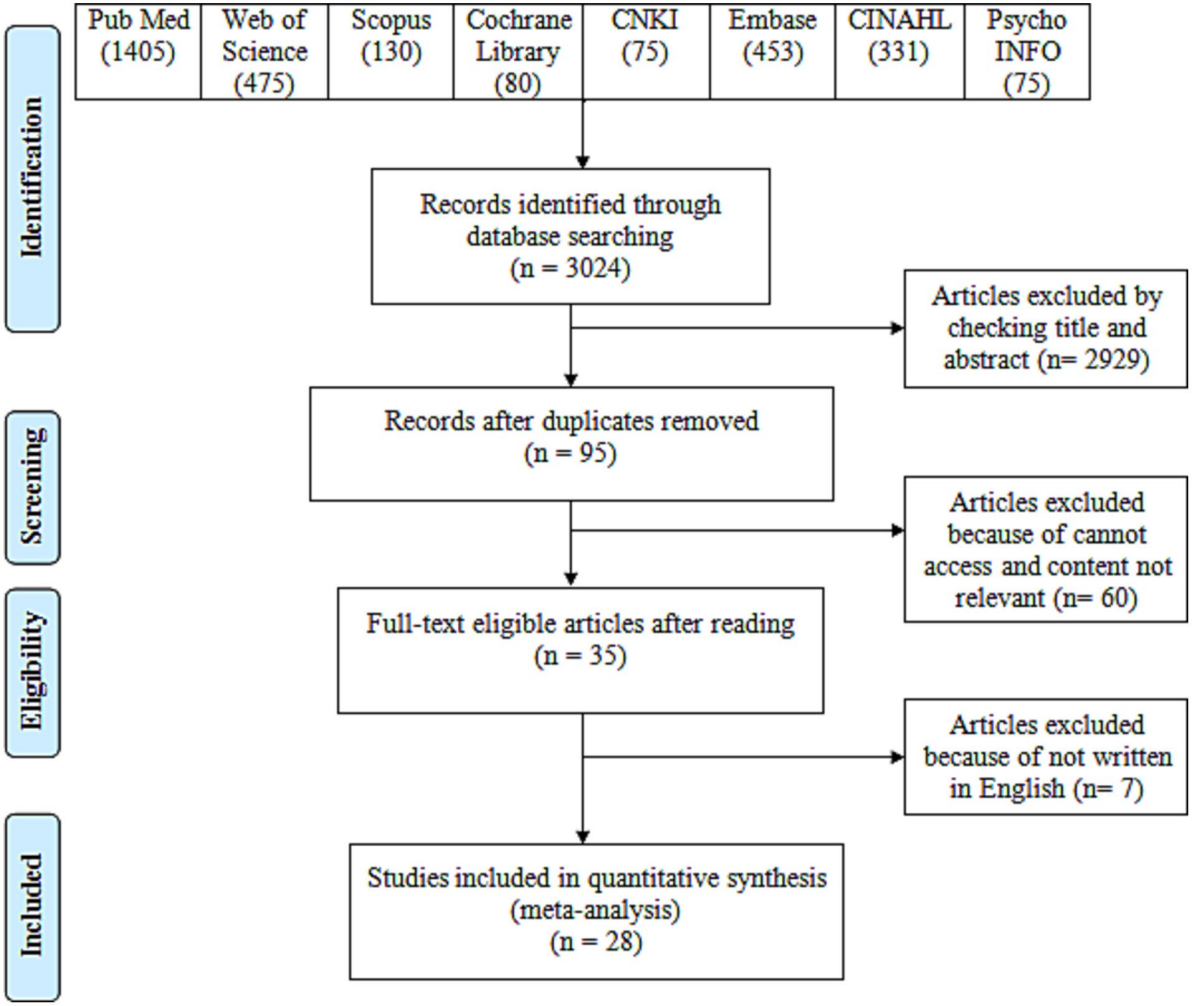
Study selection process.

We identified 3,024 articles from the seven databases (Pub Med: 1405; Web of Science: 475; Scopus: 130; Cochrane Library: 80; CNKI: 75 Embase: 453; CINAHL: 331; and PsychINFO: 75). Figure 1 shows the studies selection process. After the removal of the duplicates, 95 articles were involved in the screening of the title and abstract. Initially, the authors removed any duplicate articles from the search results, leaving them with 95 articles. The authors then screened these 95 articles by reading their titles and abstracts to see if they met the study’s inclusion criteria. The inclusion criteria refer to the characteristics or features that articles need to be relevant for the study. Out of the 95 screened articles, the authors determined that only 35 of them met the inclusion criteria. The screening process indicated that 35 articles were eligible per the inclusion criteria. Hence, 35 articles were initially selected for the full-text review. Of these 35 articles, seven did not meet the full-text review criteria and were excluded from the study. The full-text review excluded seven articles; 28 were selected for the data extraction. A total of 28 studies were considered for the meta-analysis (See Supplementary material).

### 3.1 Study characteristics

The data analysis evaluates the research outcomes based on the meta-analysis conducted. A sample of 28 articles based on mental health and interventions or social prescriptions was taken for the data collection. The study characteristics of all 28 articles were considered in the meta-analysis (see Table 1). From 2020, 12 articles were taken, six articles were taken from the year 2019, five articles were taken from the year 2021, three were from 2022, one was from 2017, and one was from 2018. Regarding the data collection, nine articles were considered experimental studies, seven articles were considered controlled studies, three of the articles conducted a pilot study, and seven articles were surveyed. At the same time, there were others with repeated measure design and treatment. In the context of the country of research, four articles were conducted in China, four were based in Iran, and five were conducted in the United States, two from Italy, the other three from the United Kingdom, and two from Sweden. In addition, the studies were conducted in South Korea, Canada, Kenya, Australia, Germany, Serbia, and France, and 18 countries study (see Table 1). Regarding gender, 20 studies were male and female, while eight were female. The study characteristics also involved the psychiatric condition of the patients, where most of the studies were based on anxiety, depression, and stress. The interventions included a mixture of natural prescriptions, social prescriptions, and cognitive-behavioral group therapy (CBGT). The studies were also characterized based on the age group, and most were conducted on youngsters.

**Table 1:** Study characteristics.

Study	Type of data collection	Country	Age group	Gender	Intervention	Marital status of participants	Psychiatric condition
Tester-Jones (2020)	Survey	18-Country	Above 18	Both	Vising nature	Married and single	Anxiety and depression
Burrai (2020)	Survey	Italy	22–73	Both	Social belongingness	Married and single	Anxiety, stress, and depression
Alipour (2020)	Experimental study	Iran	25–35	Female	Communication training	Married	Anxiety
Kim (2021)	Experimental study	South Korea	70–90	Both	Blue intervention	Married and single	Alzheimer
Sprague (2021)	Survey	United States	9–15	Both	Nature based education	Single	Quality of life
Green (2020)	Treatment	Canada	22–41	Female	CBGT	Married	Anxiety
Carnes (2017)	Survey	United Kingdom	38–78	Both	Social prescribing coordinator	Married and single	Depression and anxiety
Charbonnier (2022)	Controlled study	United States	18–25	Both	Self-help intervention	Single	Anxiety
Chory (2022)	Pilot study	Kenya	10–20	Female	Mobile intervention	Single	Anxiety
Arapovic-Johansson (2018)	Survey	Sweden	32–56	Both	Participatory, organizational intervention	Married and single	Stress
Rapisarda (2020)	Survey	Italy	32–56	Female	Inpatient services	Married and single	Burnout
Weiner (2020)	Experimental study	China	35–60	Both	CBT	Married and single	Depression
Kalmbach (2020)	Experimental study	United Kingdom	25–40	Female	CBT	Married	Insomnia
Francis (2019)	Controlled study	Australia	17–35	Female	Brief diet	Single	Depression
Wei (2020)	Repeated measure design	China	18–65	Both	Internet-based intervention	Married and single	Anxiety
Bassett (2019)	Experimental study	United States	30–60	Both	Digital intervention	Married and single	Depression
Richards (2020)	Controlled study	United Kingdom	10–50	Both	Digital intervention	Married and single	Anxiety
Alhusen (2021)	Pilot study	United States	20–30	Female	Prenatal intervention	Married, single, divorced, widowed	Depression
Pan (2020)	Experimental study	China	23–30	Both	Music intervention	Married and single	Depression
Lemay (2019)	Pilot study	United States	18–22	Both	Yoga and meditation	Single	Anxiety
Sohrabi (2022)	Controlled study	Iran	28–49	Both	Ergonomics training program	Married and single	Quality of life
Moszeik (2020)	Experimental study	Germany	19–71	Both	Yoga Nidra intervention	Married, single, divorced, and widowed	Stress
Hedblom (2019)	Experimental study	Sweden	18–50	Both	Vising nature	Married and single	Stress
Vujcic (2019)	Survey	Serbia	18–65	Both	Vising nature	Married and single	Mental wellbeing
Bayat (2021)	Controlled study	Iran	25–40	Female	Psychological	Married	Anxiety
Guerrier (2020)	Controlled study	France	55–80	Both	Music intervention	Married	Anxiety
Sun (2021)	Controlled study	China	13–85	Both	Psychological	Married, single, divorced, and widowed	Anxiety
Moghimian (2019)	Experimental study	Iran	55–65	Both	Storytelling	Married and single	Anxiety

### 3.2 Heterogeneity

The *I*^2^ index calculates the degree of absolute heterogeneity by multiplying by 100 and dividing the discrepancy between the degrees of freedom and the findings of the *Q* test by the *Q* value itself. The *I*^2^ index for the provided data set is indicated to be 95% CI. It shows that the heterogeneity is considerable in the provided data set. The value confirms that the articles used in the meta-analysis are heterogeneous. It shows that the outcomes and models used in the studies are not similar and have substantial differences (see Table 2). It rejects the assumption that the studies are homogenous and that a fixed effect meta-analysis would be a suitable measure to indicate the summary findings. Hence, random effect meta-analysis is considered more suitable for the current study.

**Table 2:** Meta-analysis results by gender.

Subgroup	*K*	SMD	95% CI	Tau^2	Tau
Both	20	0.2285	[0.1690; 0.2880]	486.54	0.8772
Female	7	0.0583	[−0.1011; 0.2178]	17.81	0.4321
Male	13	0.1987	[0.1374; 0.2600]	286.73	0.6748

K, Number of predictors; SMD, Standardized mean difference; and CI, Confidence interval.

### 3.3 Summary measures and forest plot

**Figure 2:**
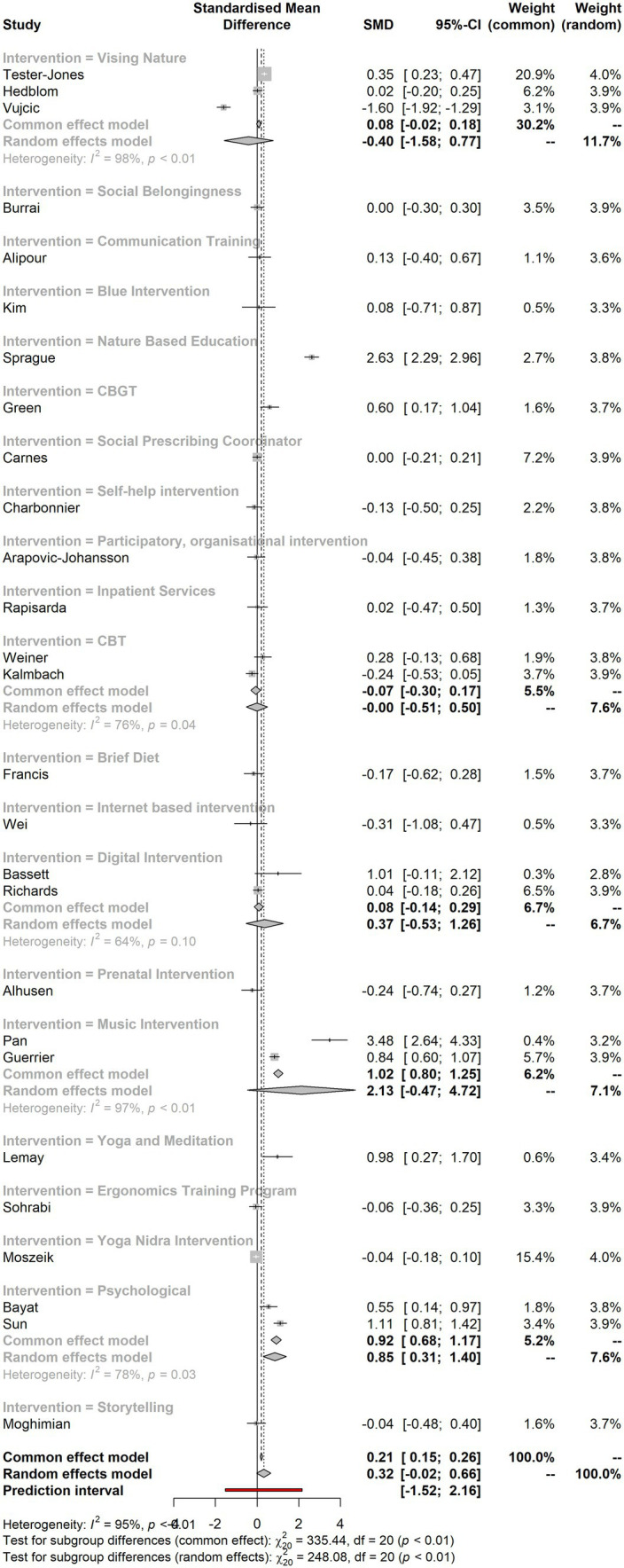
Forest plot and the summary measures for the interventions.

Figure 2 indicates the forest plot and the summary measures for the interventions. It shows that there are very few studies that have larger effect sizes. These measures tend to have strong effects, while most studies indicated small effect sizes with the two-point drop. The *I*^2^ calculation (CI 95%) also shows higher heterogeneity.

### 3.4 Effect sizes of the model for the country

**Figure 3:**
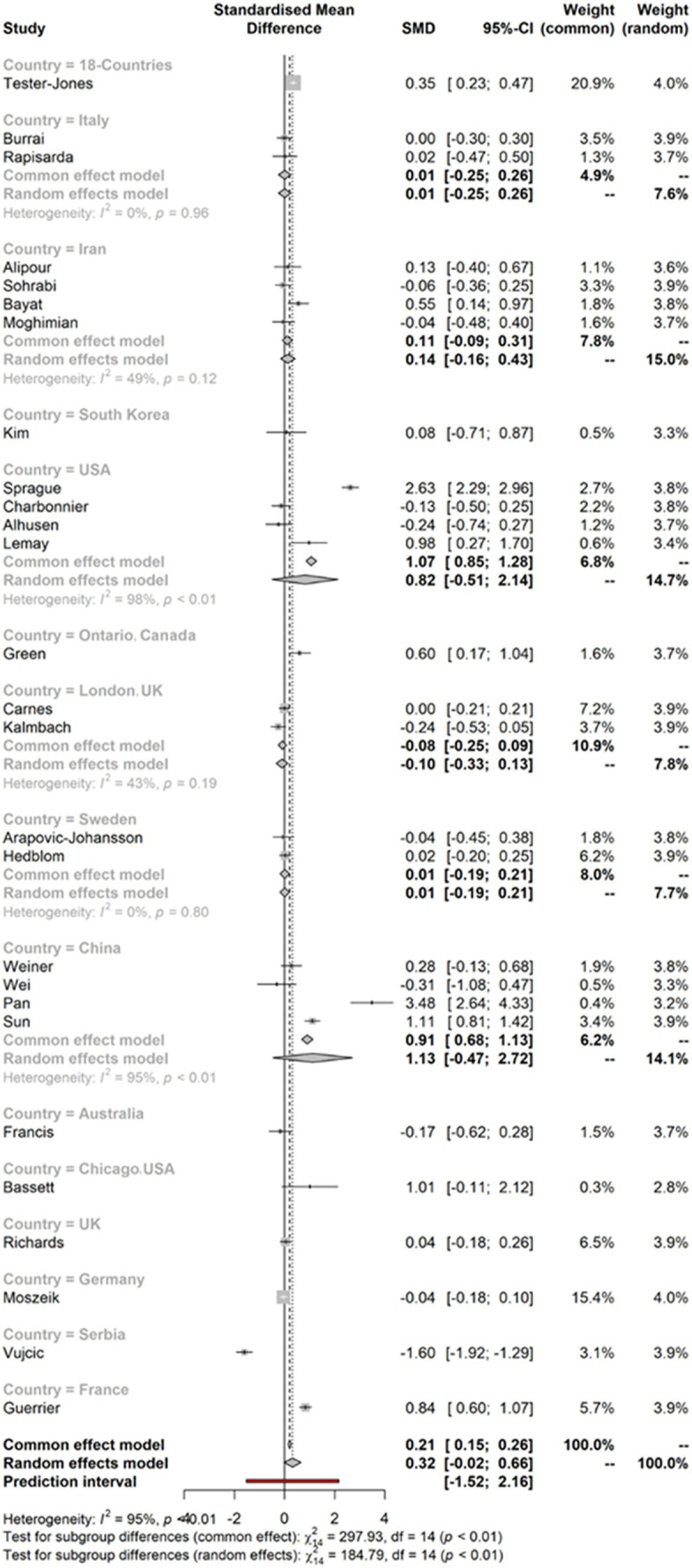
Effect sizes of the model for the country.

Figure 3 indicates the effect sizes of the model for the country. The figure shows that very few studies indicated substantial effect sizes. Most of the studies within the meta-analysis are shown to have effect sizes below two points or four points. The *I*^2^ calculation (CI 95%) also shows the heterogeneity of the data.

### 3.5 Forest plot for the type of data collection

**Figure 4:**
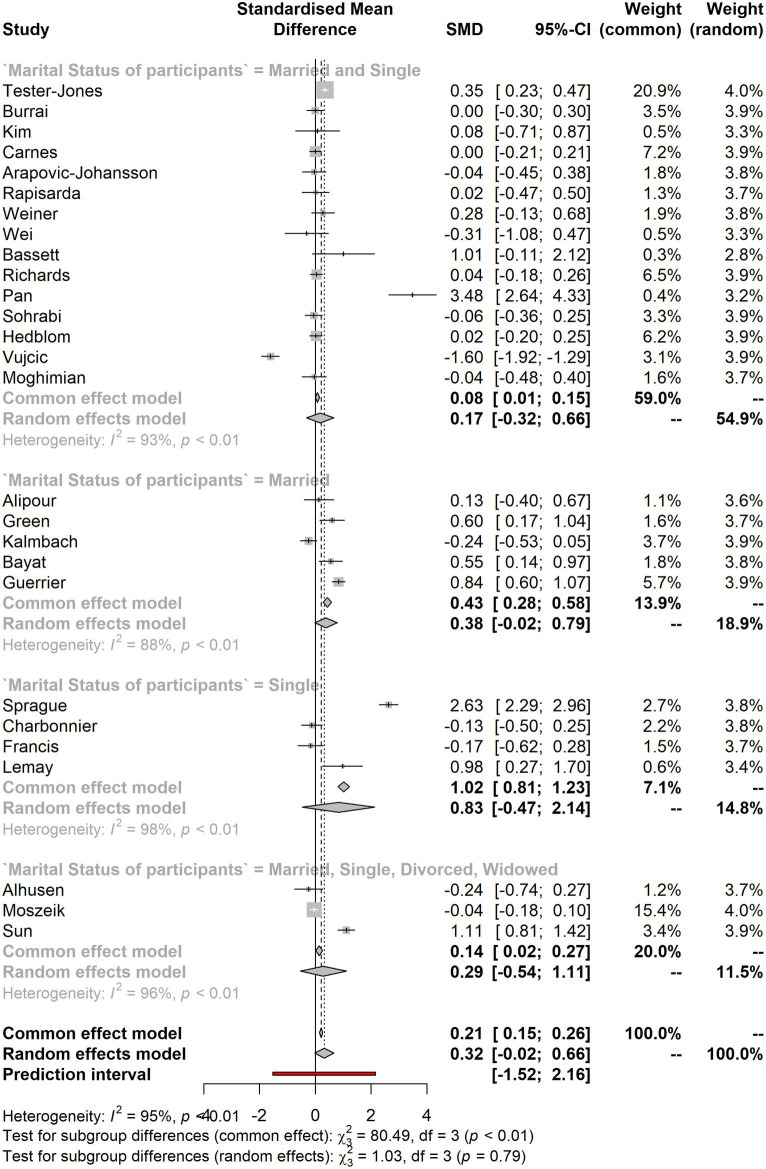
Forest plot for the type of data collection.

Figure 4 represents the forest plot for the type of data collection. The figure shows that most studies have smaller effect sizes while very few have larger and stronger ones. It shows that most of the studies fall below the point of 2.

### 3.6 Forest plot for the studies based on the year of their publication

**Figure 5:**
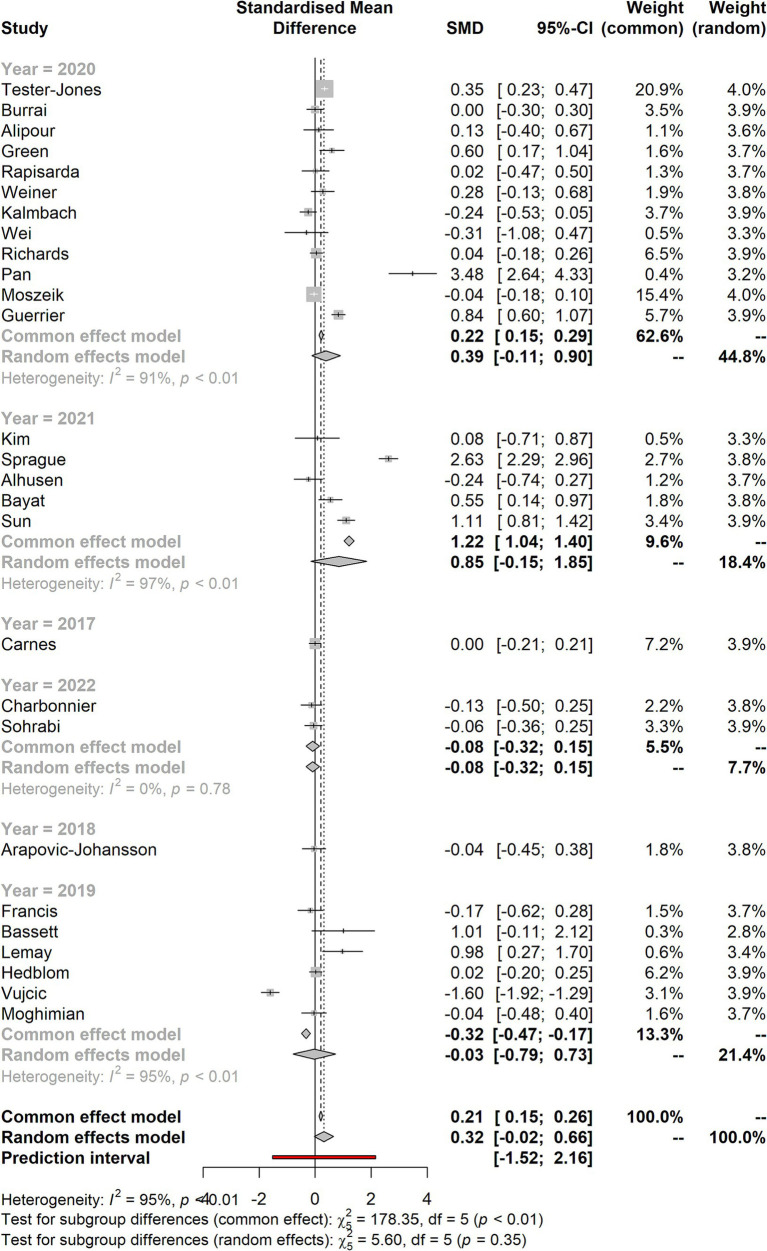
Forest plot for the studies based on the year of their publication.

Figure 5 shows the forest plot for the studies based on the year of their publication. It shows that most studies have smaller effect sizes falling below two points, whereas very few have effect sizes of less than four points. The *I*^2^ calculation (CI 95%) also shows higher heterogeneity in the data set.

### 3.7 Forest plot for the data set grouped in gender

**Figure 6:**
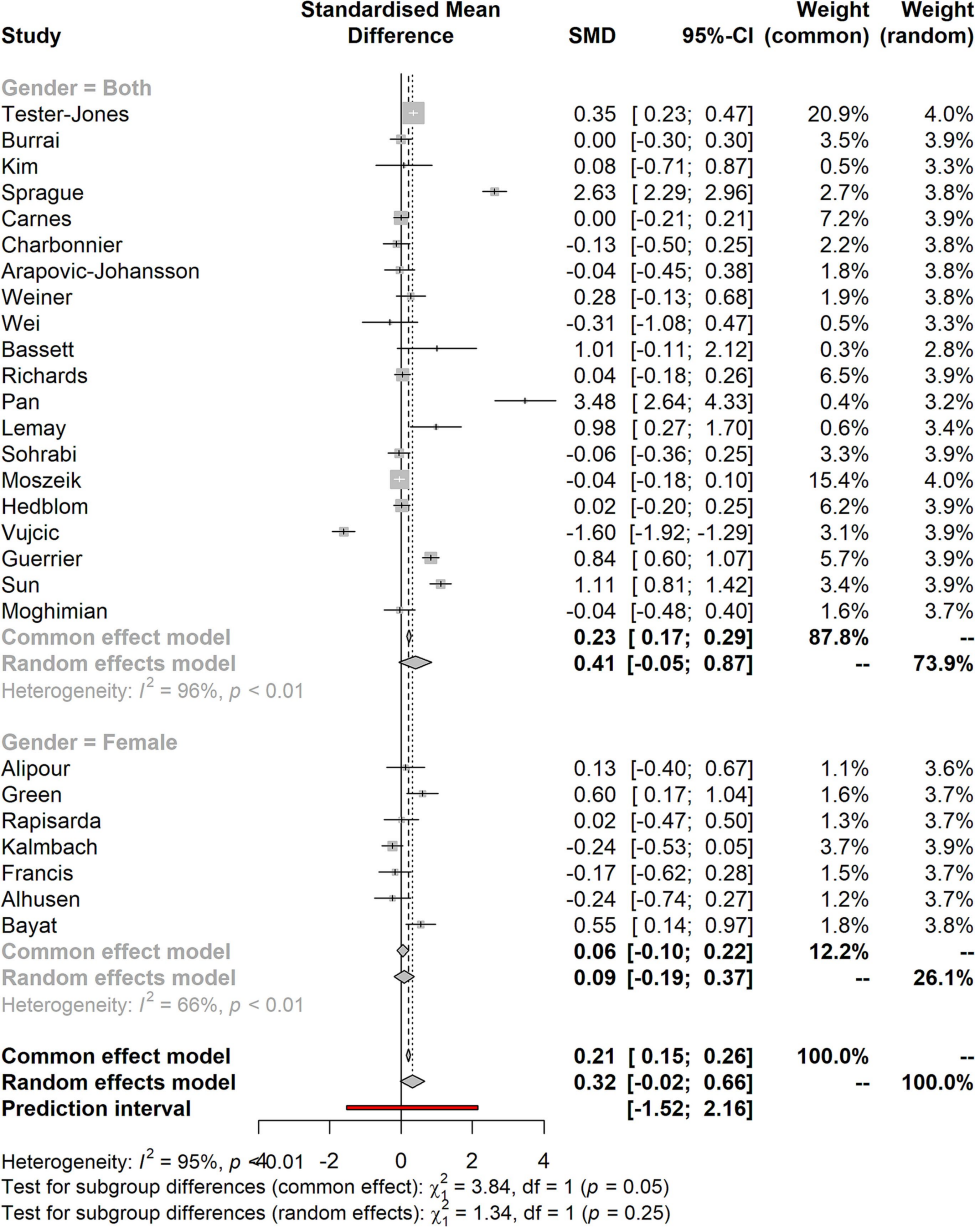
Forest plot for the data set grouped in gender.

Figure 6 shows the forest plot for the data set grouped in gender. The studies provided showed that a few of them tend to have larger effect sizes. In contrast, most studies have tiny effect sizes, falling below the two points. The *I*^2^ calculation (CI 95%) also shows the heterogeneity in the studies considered for the meta-analysis.

### 3.8 Publication bias

**Figure 7:**
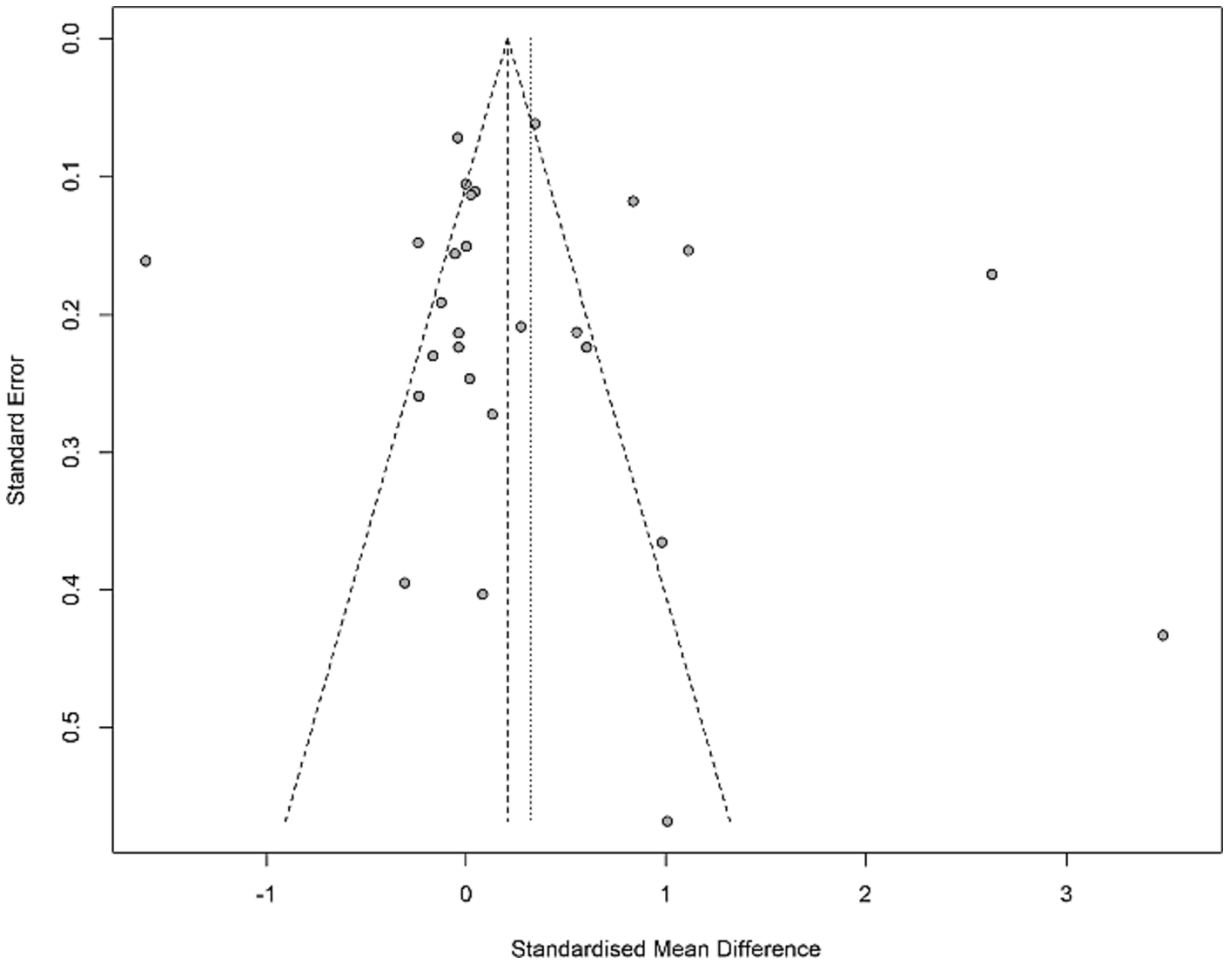
Funnel plot.

There is a high probability that certain studies that should have been included in the study were not included due to the sample bias as the studies with non-significant are not published, which can be assumed in the case of the present study. Hence, it could have affected the accuracy and generalizability of the findings (51). In addition, the exclusion of non-significant results could have affected the precision of the effect size, followed by the absence of smaller and non-significant studies that led to a wider confidence interval and less precise estimates (52). Therefore, it is critical to analyze the impact of publication bias on the study. Various techniques have been used previously, such as funnel plots, Egger’s test, and the trim-and-fill method. To assess the publication bias, funnel plots that seem symmetrical were constructed and presented in Figure 7.

Since the funnel plot seems asymmetrical, it indicates a positive sign of no publication bias in the present study. However, it is also essential to state that outliers on both sides of the plot indicate that certain studies have sample sizes that are too small, and few have a larger sample size, leading to outliers in the data. Meanwhile, these outliers can also be attributed to factors other than publication bias, such as the study’s design and quality. Therefore, these factors could have influenced the results of the meta-analysis, but there is no significant publication bias evident in the study.

### 3.9 Subgroup analysis and meta-regression

Table 2 demonstrates that nature-based social prescriptions have a moderate overall effect size (SMD = 0.2285, 95% CI [0.1690, 0.2880]) on mental health outcomes across both genders. Among females, the impact appears to be weaker (SMD = 0.0583, 95% CI [−0.1011, 0.2178]), while among males, it is moderate (SMD = 0.1987, 95% CI [0.1374, 0.2600]), falling between the effect sizes observed for the combined group and females. These results underscore the gender-specific differences in the effectiveness of nature-based social prescriptions on mental health outcomes. Furthermore, the substantial variability in effect sizes among studies for both genders, as indicated by the heterogeneity statistics, highlights the importance of considering potential moderators or subgroup differences in future research and interventions in this domain.

Table 3 presents the meta-analysis results by year, where it is evident that studies published in 2017 and 2018 did not affect mental health outcomes, as the SMD is not statistically significant and the confidence interval contains zero. However, studies published in 2019, 2020, and 2021 showed a positive effect on mental health outcomes, with SMDs ranging from −0.03 to 0.85. There was also moderate to high heterogeneity among the studies in these subgroups, as indicated by the tau^2 and tau values. The subgroup analysis for 2022 is based on only two studies, which showed no significant effect on mental health outcomes.

**Table 3:** Meta-analysis results year.

Subgroup	*K*	SMD	95% CI	Tau^2	Tau
2017	1	0	[−0.2079; 0.2079]	--	--
2018	1	−0.0372	[−0.4549; 0.3805]	--	--
2019	6	−0.0292	[−0.7887; 0.7303]	0.812	0.9011
2020	12	0.3939	[−0.1114; 0.8993]	0.7472	0.8644
2021	5	0.8499	[−0.1474; 1.8472]	1.2308	1.1094
2022	2	−0.0834	[−0.3209; 0.1540]	0	0

K, Number of predictors; SMD, Standardized mean difference; and CI, Confidence interval.

Table 4 presents the meta-analysis, including four subgroups based on marital status: married and single, married, single, and married, single, divorced, and widowed. The subgroup with the most significant number of studies (*K* = 15) is married and single, with an SMD of 0.174 and a 95% CI ranging from −0.3150 to 0.6631. The tau^2 value of 0.8649 and tau value of 0.93 suggest moderate to high heterogeneity across the studies in this subgroup. Meanwhile, referring to the subgroup married, single, divorced, and widowed have (*K* = 3) and an SMD of 0.2876 and a 95% CI ranging from −0.5374 to 1.1127. The tau^2 value of 0.5008 and tau value of 0.7077 suggest moderate heterogeneity across the studies in this subgroup. The third subgroup, single, has an SMD of 0.831 and a 95% CI ranging from −0.4733 to 2.1354; this subgroup only includes four studies. In addition, the tau^2 value of 1.7093 and tau value of 1.3074 suggest high heterogeneity across the studies. Lastly, the subgroup with the lowest heterogeneity is married to an SMD of 0.3822 and a 95% CI ranging from −0.0209 to 0.7853. The tau^2 value of 0.1725 and tau value of 0.4153 suggest low heterogeneity across the five studies in this subgroup.

**Table 4:** Meta-analysis results by marital status.

Subgroup	*K*	SMD	95% CI	Tau^2	Tau
Married and single	15	0.174	[−0.3150; 0.6631]	0.8649	0.93
Married	5	0.3822	[−0.0209; 0.7853]	0.1725	0.4153
Single	4	0.831	[−0.4733; 2.1354]	1.7093	1.3074
Married, single, divorced, and widowed	3	0.2876	[−0.5374; 1.1127]	0.5008	0.7077

K, Number of predictors; SMD, Standardized mean difference; and CI, Confidence interval.

Table 5 presents the meta-analysis results of subgroups by data collection. The survey subgroup includes seven studies and reports a small positive effect size (SMD = 0.1931) with a wide confidence interval including negative and positive effects and zero. The subgroup (data collection) has a high degree of heterogeneity, as indicated by a significant tau^2 value and a tau value of 1.2357. Meanwhile, the experimental study subgroup includes nine studies. It reports a moderate positive effect size (SMD = 0.4726) with a confidence interval that does include zero. In the third group subgroup treatment, only one study reports a moderate positive effect size (SMD = 0.6041). Therefore, based on the above results, it is evident that only the treatment group shows a statistically significant influence of the intervention on the participants’ mental health, where the intervention of green and blue nature-based social prescription may have a positive effect on mental health outcomes.

**Table 5:** Meta-analysis results data collection.

Subgroup	*K*	SMD	95% CI	Tau^2	Tau
Survey	7	0.1931	[−0.7308; 1.1170]	1.5271	1.2357
Experimental study	9	0.4726	[−0.2455; 1.1907]	1.1178	1.0573
Treatment	1	0.6041	[0.1663; 1.0419]	--	--
Controlled study	7	0.323	[−0.0626; 0.7085]	0.2417	0.4916
Repeated measure design	1	−0.3078	[−1.0819; 0.4662]	--	--
Pilot study	2	0.3452	[−0.8473; 1.5376]	0.6414	0.8009

K, Number of predictors; SMD, Standardized mean difference; and CI, Confidence interval.

### 3.10 Meta-analysis results by country

Table 6 presents the data analysis of meta-analysis by country, where the 18-countries subgroup is a standalone study that reports a small to moderate effect size (SMD = 0.3474) with a 95% confidence interval ranging from 0.2254 to 0.4694; France has SMD 0.836 with a 95% confidence interval 0.6043 to 1.0696; Canada (Ontario) has SMD 0.6041 with a 95% confidence interval 0.1663 to 1.0419, indicating that a statistically significant impact of interventions of green and blue nature-based social prescription on mental health outcomes in those countries. It also implies that intervention positively affects mental health in that studies included those studies. However, referring to other groups such as Italy, Iran, South Korea, the United States, the United Kingdom (London), Sweden, China, the United States (Chicago), the United Kingdom, Germany, and Serbia have shown moderate to higher size effects. Therefore, it can be determined that green and blue nature-based social prescriptions could be associated with improved mental health outcomes within 18 countries, including Ontario and France. Still, the strength of the association varies across different subgroups and countries. However, no other country was found to have a statistically significant association.

**Table 6:** Meta-analysis results by country.

Subgroup	*K*	SMD	95% CI	Tau^2	Tau
18-Countries	1	0.3474	[0.2254; 0.4694]	--	--
Italy	2	0.0053	[−0.2478; 0.2584]	0	0
Iran	4	0.1362	[−0.1558; 0.4281]	0.0437	0.2089
South Korea	1	0.0825	[−0.7075; 0.8726]	--	--
United States	4	0.8155	[−0.5065; 2.1375]	1.7541	1.3244
Canada (Ontario)	1	0.6041	[0.1663; 1.0419]	--	--
United Kingdom (London)	2	−0.0983	[−0.3310; 0.1344]	0.0125	0.1118
Sweden	2	0.01	[−0.1869; 0.2070]	0	0
China	4	1.1255	[−0.4695; 2.7204]	2.548	1.5962
Australia	1	−0.1666	[−0.6170; 0.2839]	--	--
United States (Chicago)	1	1.0087	[−0.1051; 2.1224]	--	--
United Kingdom	1	0.044	[−0.1750; 0.2630]	--	--
Germany	1	−0.0423	[−0.1844; 0.0999]	--	--
Serbia	1	−1.6043	[−1.9222; −1.2865]	--	--
France	1	0.8369	[0.6043; 1.0696]	--	--

K, Number of predictors; SMD, Standardized mean difference; and CI, Confidence interval.

### 3.11 Meta-analysis results by intervention

According to the Table 7 meta-analysis of interventions statistics, CBT (SMD −0.0035; 95% CI: [−0.5090; 0.5020]; Tau^2: 0.1011; Tau: 0.318), digital intervention (SMD −0.3654; 95% CI: [−0.5258; 1.2566]; Tau^2: 0.2976, Tau: 0.5455), music intervention (SMD −2.1281; 95% CI: [−0.4659; 4.7221]; Tau^2: 3.4046; Tau:1.8452), and psychological interventions (SMD −0.8529; 95% CI: [0.3051; 1.4007]; Tau^2: 0.1224; Tau: 0.3499) do not significantly impact. The other interventions (social belongingness, communication training, blue intervention, nature-based education, cognitive behavior group therapy, social prescribing coordinator, self-help intervention, participatory, organizational intervention, inpatient services, brief diet, internet-based intervention, prenatal intervention, yoga and meditation, ergonomics training program, yoga nidra intervention, and storytelling) highlighted above are significant.

**Table 7:** Meta-analysis results by intervention.

Subgroup	*K*	SMD	95% CI	Tau^2	Tau
Vising nature	3	−0.4	[−1.5824; 0.7741]	1.0699	1.0344
Social belongingness	1	0.0008	[−0.2963; 0.2980]	--	--
Communication training	1	0.1312	[−0.4029; 0.6652]	--	--
Blue intervention	1	0.0825	[−0.7075; 0.8726]	--	--
Nature based education	1	2.6262	[2.2896; 2.9628]	--	--
Cognitive-Behavioral Group Therapy (CBGT)	1	0.6041	[0.1663; 1.0419]	--	--
Social prescribing coordinator	1	0	[−0.2079; 0.2079]	--	--
Self-help intervention	1	−0.1255	[−0.5000; 0.2491]	--	--
Participatory, organizational Intervention	1	−0.0372	[−0.4549; 0.3805]	--	--
Inpatient services	1	0.0171	[−0.4657; 0.5000]	--	--
Cognitive Behavior Therapy (CBT)	2	−0.0035	[−0.5090; 0.5020]	0.1011	0.318
Brief diet	1	−0.1666	[−0.6170; 0.2839]	--	--
Internet-based intervention (specific range)	1	−0.3078	[−1.0819; 0.4662]	--	--
Digital intervention (Wider range)	2	0.3654	[−0.5258; 1.2566]	0.2976	0.5455
Prenatal intervention	1	−0.2366	[−0.7445; 0.2714]	--	--
Music intervention	2	2.1281	[−0.4659; 4.7221]	3.4046	1.8452
Yoga and meditation	1	0.9814	[0.2651; 1.6977]	--	--
Ergonomics training program	1	−0.0552	[−0.3623; 0.2519]	--	--
Yoga Nidra intervention	1	−0.0423	[−0.1844; 0.0999]	--	--
Psychological intervention	2	0.8529	[0.3051; 1.4007]	0.1224	0.3499
Storytelling	1	−0.0372	[−0.4755; 0.4011]	--	--

K, Number of predictors; SMD, Standardized mean difference; and CI, Confidence interval.

### 3.12 Meta-analysis results by psychiatric conditions

Table 8 shows the meta-analysis of the mental health condition. It shows that anxiety (SMD: 0.3955; 95% CI: [0.0901; 0.7010]; Tau^2: 0.1906; Tau: 0.4365), quality of life (SMD: 1.2846; 95% CI: [−1.3431; 3.9123]; Tau^2: 3.5679; Tau: 1.8889), and depression (SMD: 0.8407; 95% CI: [−0.4988; 2.1803]; Tau^2: 2.2055; Tau: 1.4851) does not have a significant impact while anxiety and depression (SMD: 0.3474; 95% CI: [0.2254; 0.4694]) anxiety, stress, and depression(SMD: 0.0008; 95% CI: [−0.2963; 0.2980]), Alzheimer (SMD: 0.0825; 95% CI: [−0.7075; 0.8726]), depression and anxiety (SMD: 0; 95% CI: [−0.2079; 0.2079]), burnout (SMD: 0.0171; 95% CI: [−0.4657; 0.5000]), insomnia (SMD: −0.2417; 95% CI: [−0.5333; 0.0500]), and mental well-being (SMD: −1.6043; 95% CI: [−1.9222; −1.2865]) have significant impacts.

**Table 8:** Meta-analysis results by psychiatric conditions.

Subgroup	*K*	SMD	95% CI	Tau^2	Tau
Anxiety and depression	1	0.3474	[0.2254; 0.4694]	--	--
Anxiety, stress, and depression	1	0.0008	[−0.2963; 0.2980]	--	--
Anxiety	10	0.3955	[0.0901; 0.7010]	0.1906	0.4365
Alzheimer	1	0.0825	[−0.7075; 0.8726]	--	--
Quality of life	2	1.2846	[−1.3431; 3.9123]	3.5679	1.8889
Depression and anxiety	1	0	[−0.2079; 0.2079]	--	--
Stress	3	−0.0244	[−0.1396; 0.0909]	0	0
Burnout	1	0.0171	[−0.4657; 0.5000]	--	--
Depression	5	0.8407	[−0.4988; 2.1803]	2.2055	1.4851
Insomnia	1	−0.2417	[−0.5333; 0.0500]	--	--
Mental well-being	1	−1.6043	[−1.9222; −1.2865]	--	--

K, Number of predictors; SMD, Standardized mean difference; and CI, Confidence interval.

## 4 Discussion

Green prescription is becoming more popular in clinical and public health settings, especially for patients routinely visited for psychological disorders (53). Rapid population aging worldwide has elevated older people’s mental health to the forefront of public health concerns. The adverse effects of poor mental health in old age are substantial, extending to diminished physical and cognitive capacity and increased risk of illness and death. The presence of water features or other “blue spaces” in a neighborhood has been linked to better mental health. However, a small body of research still supports the promise of neighborhood blue spaces to improve seniors’ mental health (54). Studies on the positive effects of time spent in nature for health maintenance and improvement are increasing worldwide (55). Our finding shows that the natural prescriptions significantly influence stress outcomes. It is found that there is a statistically significant effect of nature-based social prescriptions on mental health outcomes for the “both” subgroup, which includes both males and females, with an effect size of 0.2285. It has also determined that nature-based social prescriptions positively affect mental health outcomes for both males and females, although the effect may be more substantial for males. However, the effect may vary depending on individual differences or other factors not controlled for in the meta-analysis. It has also indicated that the effectiveness of green and blue nature-based social prescriptions for mental health could increase over time. The results are consistent with the study (56), which used a systematic review and meta-analysis to assess the effects of outdoor activities rooted in nature on both physical and mental health. A total of 50 studies were found after screening 14,321 records. The current study included 16 research studies that were based on uncontrolled before-and-after investigations, 18 studies that were controlled, and 16 studies that were based on randomized controlled trials. For controlled and uncontrolled studies, the biasness risk for randomized control trials (RCT) was low to moderate and moderate to high. Nature-based interventions (NBIs) were beneficial in enhancing depressive mood and lowering anxiety levels, according to the results of the random effect meta-analysis for RCTs. Our meta-analysis statistics in the country perspective show a small to moderate effect size (SMD = 0.3474) with a 95% confidence interval ranging from 0.2254 to 0.4694; France has SMD 0.836 with a 95% confidence interval of 0.6043 to 1.0696; and Ontario has SMD 0.6041 with a 95% confidence interval 0.1663 to 1.0419, indicating that a statistically significant impact of interventions of green and blue nature-based social prescription on mental health outcomes in those countries.

Spending time in a natural setting, such as a forest, has been shown to positively affect mental and physical health, making forest therapy an increasingly popular option for those seeking stress relief and improved quality of life (57). Novel businesses like “green cafés” and nature-inspired arts activities have evolved to help people during and after the epidemic. These programs establish green social prescribing in the local community (58). In contrast to public parks and other urban green spaces, home gardens are less frequently acknowledged as a source of therapeutic benefit. Nonetheless, home gardens may improve both mental and physical health (59). Gardening, conservation activities, and ecotherapy are all examples of nature-based social prescribing that have been demonstrated to increase social connectivity, enhance social networks, decrease stress, and boost health and well-being (60). Participating in activities in natural surroundings encourages well-being and health-promoting behaviors such as physical activity and social interaction. Compared to those who do not visit green spaces, those who do at least once a week are more likely to get the appropriate amount of exercise (61). People with lower socioeconomic status and poorer levels of well-being have been shown to gain the most from engaging with natural areas (62). Numerous volunteer programs provide resources and guidance to groups or individuals interested in doing hands-on work in the outdoors. Creation and restoration of natural ecosystems, upkeep of outdoor facilities like parks, pathways, and trails, and construction of physical elements in the natural environment are all examples of the kinds of things that go under the umbrella term “landscape architecture” (63).

Much evidence shows that spending time in green spaces and outside doors positively affects one’s health and happiness. Community gardens have both physical and social advantages which enhances psychosocial health and well-being (64–67). According to Japanese research, senior citizens who garden at home report higher satisfaction levels and better health due to the activity (68). Human-nature connections have been the subject of philosophical contemplation in China for millennia. Humans have been singled out for using plants and the environment as therapeutic therapy for better mental and physical health. Our findings also aligned with the above results and reported that Italy, Iran, South Korea, the United States, Sweden, China, the United Kingdom, Germany, and Serbia have shown moderate to higher size effects. The ecological knowledge of the connection between human health and the environment may be found in the Taoist culture of China’s pre-Qin dynasty and has further indicated that the connection with nature is widely indicated to alleviate psychological stress (69). From a human well-being standpoint, trees significantly improve the quality of life. The health of a community benefits from the presence of trees and other plants because they help regulate the flow of snow and rainfall, improve air quality, lower temperatures in the summer, and increase biodiversity (59, 60). The influence of the natural environment on stress response has been examined in 26 studies. Eighteen studies received a moderate quality rating, four received a low-quality rating, and four received a high-quality rating. The meta-analysis outcomes reflected that seated relaxation and walking in the natural atmosphere increase the heart rate and reduce stress. It has, therefore, indicated that natural-based prescriptions are suitable for reducing individual stress. Sedentary habits and poorer outcomes for women’s mental health have been linked to urbanism. It is generally accepted that proximity to natural places may increase physical activity and improve psychological health in urban settings. However, there is a lack of information regarding how women utilize natural environments to improve their health and well-being in daily settings (70).

General medicine doctors are increasingly prescribing the “green prescription” to inactive patients. Increased morbidity and death associated with obesity are strongly linked to inactivity. Traditional medical treatments, especially those for non-communicable illnesses and mental health disorders, may be complemented with green prescriptions tailored to meet individual patients’ needs (71). Our intervention-based meta-analysis statistics show that blue nature intervention and nature-based education significantly impact mental health outcomes. Ecological psychology, which has its roots in humanity’s estrangement from the natural world, seeks to repair that breach by encouraging people to spend more time in the outdoors; it also emphasizes the therapeutic value of natural ecosystems and uses nature’s recuperative powers to treat mental illness and spur personal growth (72). Many tiers of nature access and engagement might serve as a social prescription. Various social, economic, and environmental elements are now understood to affect people’s health. Similar to social prescriptions, green prescriptions seek to facilitate healing via contact with natural environments. Similarly, Nguyen et al. (73) has provided a review and meta-analysis of appraised evidence on the nature of prescription’s effectiveness on different health outcomes. In this way, the study has determined the factors critical for natural prescription success. It was indicated that the natural-based prescriptions have significantly impacted the participants’ stress and blood pressure levels. It also has a moderately higher impact on depression and anxiety scores.

The outcome of the current research is primarily consistent with the previous research. Therefore, it is indicated that nature-based prescriptions significantly impact mental health outcomes. Psychologists are recommended to use these interventions for improved results in the patient’s mental health. Cost-effective, simple, low-risk, and entertaining nature-based activities are gaining popularity as preventative and therapeutic interventions. They may significantly impact the lives of those who do not have much chance to interact with the natural world regularly (74). Artistic, academic, recreational, and ecological pursuits are all fair game for social prescribing. Community gardens, parks, forests, and trails are all examples of current social prescriptions that include “nature,” an approach that builds on a history of treatments that includes terms like “green care,” “horticultural therapy,” and “nature-aided therapy” (75). These include anything from community-wide initiatives to improve health, such as green gyms or gardens, to targeted therapeutic programs for specific populations, such as care farms, walk-and-talk therapy, or horticultural therapy. More people might exercise and spend more time outside if parks were made more easily accessible, and park-goers would be happier. In addition to providing fresh air and exercise, parks also help people socialize and escape their shells (76, 77).

## 5 Conclusion

It has been found that eco-friendly activities like gardening, exercise outside, and nature-based treatment significantly enhance the mental health of individuals, including those who already have mental health problems. Evidence is mounting that gardening may help keep you from falling by promoting healthy gait and balance and can also help ward against dementia and slow mental decline (69). The reduction in physiological stress markers detected by diurnal cortisol profiles is consistent with residents with more plants in their front gardens and reports of reduced stress. More greenery in parks has been demonstrated to benefit mental health, and older persons’ access to physical space, as well as exposure to gardens, have both been linked to improved mental health. According to the study, people’s symptoms of stress, anxiety, and depression can be considerably diminished by using these natural therapies. It suggests that spending time in nature can be a helpful addition to traditional mental health interventions and can help improve mental health outcomes for individuals struggling with these common mental health conditions. Overall, the conclusion of the meta-analysis supports the idea that incorporating nature-based interventions into mental healthcare plans can effectively complement traditional therapies and improve patient outcomes. The study highlights the potential of nature to promote mental health and well-being by providing robust evidence for the benefits of nature-based social prescription.

## 6 Study implications

The study findings have important implications for healthcare providers, policymakers, and individuals seeking to improve their mental health. These findings suggest that nature-based interventions could be integrated into mental health treatment plans to complement traditional therapies, such as medication and talk therapy. It could lead to better patient outcomes and potentially reduce healthcare costs by offering a natural and affordable alternative to medication. In addition, these findings suggest that investing in nature-based interventions could effectively improve population-level mental health outcomes. It could involve funding programs that provide access to nature-based activities or integrating nature-based interventions into existing mental health policies. For individuals seeking to improve their mental health, these findings suggest that spending time in nature, such as gardening or taking walks, could be a helpful addition to their self-care routines. It could be particularly beneficial for individuals who may not have access to traditional mental healthcare services or may be hesitant to seek help for mental health concerns. Nonetheless, more research is required to understand better how NBSP enhances mental health and to pinpoint the interventions that are most successful for different populations. It could involve investigating the specific aspects of nature-based interventions that are most beneficial, such as the level of physical activity or the type of natural environment, as well as identifying populations that may benefit most from these interventions, such as individuals with specific mental health conditions or those from disadvantaged communities.

## 7 Study limitations

We conducted systematic reviews with the primary objective of analyzing previous research through a meta-analysis to ascertain the overall effect size of NBSP’s outcomes for mental health. We used MeSH in our search strategy. We excluded animal therapy-based studies and only included ecotherapy, green therapy, green exercise, activities performed or related to green spaces, or green prescriptions on mental health outcomes. As a result, it is possible that we overlooked particular research that highlighted unusual nature-based treatments. Furthermore, it is possible that we overlooked pertinent research published in other languages and added bias as a result of missing data because we only considered research published in English and peer-reviewed.

## Data availability statement

The original contributions presented in the study are included in the article/Supplementary material, further inquiries can be directed to the corresponding author.

## Author contributions

RM: Conceptualization, Data curation, Formal analysis, Methodology, Resources, Software, Validation, Writing - original draft. LY: Conceptualization, Methodology, Supervision, Writing - review & editing. ZS: Formal analysis, Validation, Software, Writing - original draft. MY: Data curation, Resources, Writing – review & editing. MS: Data curation, Resources, Writing – review & editing. All authors contributed to the article and approved the submitted version.
